# A Study of Vitamin D Status and Its Influencing Factors among Pregnant Women in Szeged, Hungary: A Secondary Outcome of a Case–Control Study

**DOI:** 10.3390/nu16101431

**Published:** 2024-05-09

**Authors:** Evelin Polanek, Anita Sisák, Regina Molnár, Zsuzsanna Máté, Edina Horváth, Gábor Németh, Hajnalka Orvos, Edit Paulik, Andrea Szabó

**Affiliations:** 1 Department of Public Health, Albert Szent-Györgyi Medical School, University of Szeged, 6720 Szeged, Hungary; 2 Doctoral School of Interdisciplinary Medicine, University of Szeged, 6720 Szeged, Hungary; 3 Department of Family Medicine, Albert Szent-Györgyi Medical School, University of Szeged, 6725 Szeged, Hungary; 4 Department of Obstetrics and Gynecology, Albert Szent-Györgyi Medical School, University of Szeged, 6725 Szeged, Hungary

**Keywords:** vitamin D, deficiency, insufficiency, pregnancy, dietary supplement, food frequency questionnaire

## Abstract

Adequate vitamin D (VD) intake during pregnancy is needed for fetal development and maternal health maintenance. However, while there is no doubt regarding its importance, there is not a unified recommendation regarding adequate intake. The main aim of our study was to measure the VD serum level of studied women, together with its potential influencing factors: demographic (i.e., age, level of education, relationship status and type of residence), conception and pregnancy related factors. Results are based on secondary data analyses of a retrospective case–control study of 100 preterm and 200 term pregnancies, where case and control groups were analyzed together. Data collection was based on a self-administered questionnaire, health documentation, and maternal serum VD laboratory tests. VD intake was evaluated by diet and dietary supplement consumption. According to our results, 68.1% of women took some kind of prenatal vitamin, and only 25.9% of them knew about its VD content. Only 12.1% of included women reached the optimal, 75 nmol/L serum VD level. Higher maternal serum levels were associated with early pregnancy care visits (*p* = 0.001), assisted reproductive therapy (*p* = 0.028) and advice from gynecologists (*p* = 0.049). A correlation was found between VD intake and serum levels (*p* < 0.001). Despite the compulsory pregnancy counselling in Hungary, health consciousness, VD intake and serum levels remain below the recommendations. The role of healthcare professionals is crucial during pregnancy regarding micronutrients intake and the appropriate supplementation dose.

## 1. Introduction

During the new millennium, vitamin D (VD) consumption has become the focus of attention in many nutrition-related research fields. Recent research has revealed that low VD levels are still a global public health issue, and not only affecting the elderly population: nearly 50% of the global population has VD insufficiency (serum VD level below 50 nmol/L) [1,2,3]. The prevalence of VD deficiency (serum VD level below 30 nmol/L) ranges from 5.9% to 20% around the world, affecting developed and developing countries [4]. The occurrence of low VD status varies by ethnicity, gender, geographical area and seasonal variations [5,6], and depends on diet, air pollution, sun avoidance (clothing practice, sunscreen use), comorbidities, drug use, food fortification policy or a shift towards indoor work [7]. The optimal serum VD level, however, is not defined uniformly. According to international recommendations, a VD level above 50 nmol/L is considered to be the sufficient level. This threshold is based on data of several large studies [8], as bone manifestations of VD hypovitaminosis can appear below this level. However, the studies also conclude that the optimal level should be defined at a higher, 75 nmol/L level [9], as below this cut-off point bone mineralization is impaired and the parathyroid hormone is elevated [10]. Other studies suggest that a 75–125 nmol/L serum level is necessary in order to achieve health benefits, but above this range no excess advantage is observed [11]. According to the Hungarian recommendations, the sufficient and the optimal levels are not separated, and the recommended normal range is 75–125 nmol/L [12]. Different health conditions—including pregnancy—can be identified as a higher risk for VD deficiency [13]. Low VD status is very common among pregnant women worldwide, especially in Middle Eastern and Asian countries, where the prevalence of VD insufficiency is 38–90% [14], but the prevalence is also high in North America (24–33%) and Europe (20–77%) as well [15].

During pregnancy, VD increases calcium absorption, enhances placental calcium transport and plays a very important role in maternal and fetal immune regulation. Several studies have shown potential correlations between the serum level of VD and maternal infections, preeclampsia, gestational diabetes mellitus, low-birth weight, preterm birth and malformations [15,16,17,18]. Therefore, adequate VD intake is necessary due to its important role in fetal development and maternal health maintenance. However, it is obvious that maternal VD intake levels should be increased to achieve sufficient levels, and there are no uniform recommendations; it varies worldwide according to scientific societies. International recommendations generally propose 10 or 15 μg (400–600 IU) VD daily (with outliers of 200 or 800 IU), with a tolerable upper intake of 100 μg per day (4000 IU) [19,20,21]. During pregnancy, the same VD intake dose is advised as for the general population, despite several recent meta-analysis and review articles concluding that the prevention of negative maternal and perinatal outcomes requires higher supplemental doses, and that the official recommendations should be updated [21,22,23]. The Hungarian consensus [12] does not differentiate between adults and pregnant women, and the daily recommended intake is 50 μg (2000 IU) during the UV-B radiation-free period with the same tolerable upper intake levels as stated in international guidelines. VD intake can be managed in three main ways: by consuming food rich in VD; via ultraviolet (UV) radiation; and by using dietary supplements containing VD [24].

A very important VD source is a diverse and healthy diet. Sea fish, egg yolks, mushrooms, dairy products, liver and foods fortified with VD (e.g., margarine, juices, dairy products, etc.) are good sources of VD [25]. However, the required level is practically impossible to fulfil through diet alone, and the mean daily dietary intake is generally less than 5 μg in most countries [26]; therefore, the use of VD-containing dietary supplements should be considered. Several international studies have evaluated the dietary supplement consumption of pregnant women. Jun et al. [27] found that 77% of American pregnant women used some kind of dietary supplementation during their pregnancy. According to a Swedish cohort study of pregnant women’s vitamin intake, in 78% of cases any kind of vitamin intake, and in 43% specifically VD intake, was observed [28].

The main aim of our study was to measure VD serum levels among pregnant women who delivered their baby in Szeged (Hungary), and its potential influencing factors, such as age, demographic characteristics, conception and pregnancy-related factors and VD intake with diet and/or supplementation.

## 2. Materials and Methods

### 2.1. Study Design

The “Quantifying MAternal Non-Obstetrical Risk Factors for Preterm Birth—Retrospective and Prospective Study” (MANOR study) was performed as a collaboration of the Department of Obstetrics and Gynecology and the Department of Public Health, University of Szeged. The present results are based on the data of the retrospective case–control branch of the study performed among women delivering in 2019 between March and December.

### 2.2. Participants

The total number of newborns at the university hospital during the study period (March–December) was 2352, out of which 277 were preterm infants. Excluding multiple pregnancies, the remaining number of eligible infants was 191. Preterm pregnancy was defined as delivery before 37 weeks of gestational age; those cases where delivery happened at the 37th week or later were considered to be term pregnancies. Participation was offered for all women who had preterm delivery during the study period; however, due to the sensitive nature of the issue, 100 preterm deliveries were ultimately included in the present analyses; these mothers made up the case group. After every included preterm birth, two term deliveries were included as controls, adjusted to maternal age and date of delivery. Multiple pregnancy was an excluding criterion in both groups. Altogether, 300 women were included in the study, 100 preterm and 200 term pregnancies. As a preliminary analysis, we compared the case and the control groups regarding VD intake and maternal VD serum levels. No significant differences were detected between the groups, and therefore in the present analysis both cases and controls were analyzed together in a secondary outcome model focusing on the possible correlations between demographic, conception and pregnancy-related characteristics and VD intake and VD serum levels of included women.

### 2.3. Measures

Data collection was based on a self-administered questionnaire, health documentation and maternal serum and umbilical cord-blood VD laboratory tests. General, sociodemographic, lifestyle, conception and previous and current pregnancy-related questions were analyzed.

Maternal sociodemographic status included maternal age, educational level, type of residence and relationship status (single or in relationship). We also asked whether the present pregnancy was the women’s first pregnancy; whether she planned the pregnancy; and whether she had any preceding pregnancy complications, including spontaneous abortion (miscarriage) or preterm delivery. The use of contraceptives before present pregnancy, mode of conception and potential infertility treatment at present pregnancy or assisted reproductive treatment (ART) were investigated, too. The week of the first visit to pregnancy care was also evaluated; nonetheless, we asked the women whether they received any information about a healthy lifestyle during pregnancy, and if yes, what was the source of the information. A question about more conscious dietary habits during pregnancy was also inserted into the questionnaire.

VD intake was evaluated through intake in the diet, prenatal multivitamins or other VD-containing dietary supplement intake (VD alone or in combination with other minerals, or plain multivitamins formulated for the general adult population). To evaluate the maternal dietary VD intake, we used a validated food frequency questionnaire (FFQ) established by Bärebring et al. [29]. The FFQ contained questions about the main sources of dietary VD, namely sea fish, milk, yoghurt/sour-milk and margarine consumption, their frequency of consumption, and in case of milk and yoghurt, their type (regarding fat content). The VD content per serving of the possible answers was given in the original article; the maximum intake was calculated to be 14.95 μg/day. Our questionnaire consisted of separate questions regarding prenatal vitamins, and any other VD-containing products or multivitamins, whether the pregnant women used to consume them during pregnancy, and if yes, what was the name and dosage of the product. The VD content of all mentioned products was manually searched and calculated by the help of official websites of manufacturers. If the responder stated that she had not used a VD-containing food supplement, but elsewhere in the questionnaire (questions not used in this article) mentioned a product that actually contained VD, then we also calculated that VD amount in the supplemental VD intake.

Blood samples were collected from mothers right after their labor. VD (25-OH-D3 vitamin) was measured by chemiluminescent microparticle immunoassay at the Department of Laboratory Medicine, University of Szeged.

In this article, we followed the classification of the US Institute of Medicine, as VD deficiency is stated to be below 30 nmol/L, and inadequacy or insufficiency is defined as between the 30 and 50 nmol/L cut-off points [30].

### 2.4. Statistical Analysis

Characteristics of the study population were evaluated by descriptive statistics (number and percentages of responses in case of categorical variables, mean and/or median and interquartile range for continuous variables), and the correlations between VD intake and demographic, conception- and pregnancy-related factors were assessed with chi-squared tests, univariable and multivariable logistic regression analysis, calculating odds ratios (OR), adjusted odds ratios (AOR) and 95% confidence intervals (95% CI). The multivariable analyses included independent variables with significant univariable associations. Due to the non-normal data distribution examined using the Kolmogorov–Smirnov test, we used Mann–Whitney U and Kruskal–Wallis tests when statistical analyses were performed with continuous variables regarding VD intake and serum VD levels. To evaluate the correlations, we used Spearman’s tests. We considered a result significant if *p* < 0.05, in the case of each statistical method. Statistical analysis was performed by using IBM SPSS Statistics 29.0 program.

### 2.5. Ethics

The study protocol was approved by the Regional and Institutional Human Medical Biological Research Ethics Committee of the University of Szeged, Hungary (approval reference number: 4419). Participation was voluntary, and written informed consent was obtained from each participant.

## 3. Results

### 3.1. Study Population Characteristics

Table 1 shows demographic, conception-related and pregnancy-related characteristics of the study population. The average maternal age of the included women was 32.28 years; the oldest mother was 45, and the youngest 18 years old. Maternal age was significantly associated with educational level (*p* < 0.001), use of contraceptives (*p* = 0.036), infertility treatment (*p* = 0.001), planned pregnancy (*p* < 0.001), first pregnancy (*p* < 0.001) and timing of first visit to pregnancy care (*p* < 0.001). Maternal educational level was significantly associated with maternal age (*p* < 0.001), use of contraceptives (*p* = 0.002) and planned pregnancy (*p* < 0.001). The mothers’ relationship status and type of residence were not associated with any other characteristics.

More than 40% of the included pregnancies were first pregnancies. Overall, 41.5% of included women had university or even PhD degrees, and 8.5% of them had finished only eight classes or fewer. The majority of women (97.6%) lived in some kind of relationship. Women received information related to lifestyle during pregnancy mostly from midwifes, gynecologists, internet and family members or friends, while other information sources—like books, magazines, TV and radio—were less frequently used.

### 3.2. Dietary VD Intake

Three quarters (75.7%) of women reported additional attention to a healthy diet during pregnancy. More than half of the included women did not eat sea fish during their pregnancy, and only 8.2% of consumers ate it at least twice a week. Overall, 66.3% drank milk daily, mostly medium fat (2.8%) or low fat (1.5%) milk; 51.5% ate some kind of fruit- or vanilla-flavored yoghurt daily. Only 30.2% used margarine daily as a spread, and 32.1% never used it [30]. Calculated dietary VD intake is shown in Table 2. In order, milk, fish and then margarine served as sources of VD, with negligible amounts in yoghurt. The mean total daily dietary VD intake was 2.08 μg.

Total dietary VD intake was not associated with any maternal personal, conception- or pregnancy-related characteristics.

### 3.3. Supplemental VD Intake

During pregnancy, 205 (68.1%) women took some kind of prenatal vitamins (Table 3). In the case of preceding infertility treatment (*p* = 0.022), conception with ART (*p* = 0.020) and early presentation at pregnancy care (*p* = 0.020), significantly higher frequent prenatal multivitamin consumption was observed. In addition to prenatal vitamins, 66 women (22.4%) took other VD-containing dietary supplements. Overall VD supplemental consumption (Table 4) was significantly associated with maternal age group (*p* = 0.047), higher education level (*p* = 0.049), preceding infertility treatment (*p* = 0.032), planned pregnancy (*p* = 0.039), first pregnancy (*p* = 0.034), earlier appearance at pregnancy care (*p* = 0.018) and advice regarding pregnancy (*p* = 0.032). Crude odds ratios of univariable logistic regression analyses are shown by Table 3 and Table 4. Women aged between 25 and 34 years had significantly higher odds of prenatal vitamin consumption compared to the younger age group. Preceding infertility treatment, conception with ART and early presentation at pregnancy care significantly increased the likelihood of prenatal vitamin consumption (Table 3). In the case of multivariable analysis, only the preceding infertility treatment remained significant (AOR: 3.18; 95% CI: 1.16–8.75; *p* = 0.025). The univariable analysis of overall VD supplementation showed significantly higher odds in women aged 25–34 years, preceding infertility treatment, planned pregnancy, first pregnancy, early presentation at pregnancy care and receiving advice regarding pregnancy (Table 4). The multivariable model confirmed the significant effect of preceding infertility treatment (AOR: 5.58; 95% CI: 1.26–25.00; *p* = 0.025), early presentation at pregnancy care (AOR: 2.44; 95% CI: 1.10–5.42; *p* = 0.028) and having a first pregnancy (AOR: 2.18; 95% CI: 1.10–4.32; *p* = 0.026).

Only 25.9% of those women who took prenatal vitamins knew that they contained VD. Knowledge about the presence of VD was significantly associated with information received from the internet (*p* = 0.006) and previous preterm birth (*p* = 0.034), and a non-significant correlation was found with infertility treatment (*p* = 0.061) and assisted reproductive techniques (*p* = 0.068).

### 3.4. Total VD Intake

Total VD intake results are shown in Table 5. All the reported prenatal vitamins contained VD; the average daily intake was 9.22 μg (SD = 11.21 μg). The daily average intake with other dietary supplements was 7.97 μg (SD = 20.18 μg). Overall, 77.6% of women took some kind of VD-containing dietary supplement, in the form of prenatal vitamins, other vitamins or both, and their average daily VD supplementation was 17.19 μg (SD = 22.45 μg) [31]. The average total (dietary plus supplemental) VD intake was 19.27 μg/day (SD = 22.44), and 44.5% (*n* = 134) of women reached the international, 15 μg/day intake recommendation. The lower band of the Hungarian recommendation of an intake of at least 37.5 μg/day was reached by only 36 participants (12.0%). A significantly higher total VD intake was associated with higher education level (*p* = 0.026), use of contraceptives (*p* = 0.007) and conception with ART (*p* = 0.034).

### 3.5. Serum VD Level

**Figure 1:**
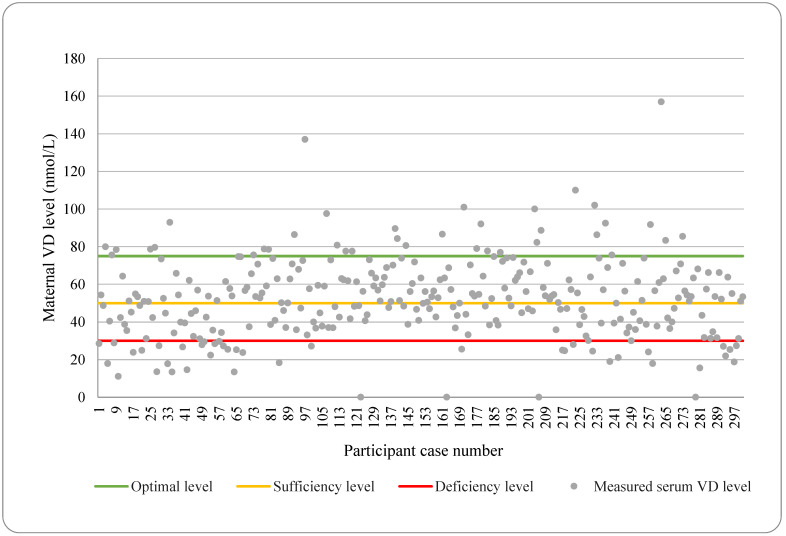
Serum maternal vitamin D (VD) level among included women.

The average maternal serum VD level was 52.81 nmol/L, and the median level was 52.4 nmol/L [31]. According to our results, 39 women (13.1%) remained under the minimal deficiency level (30 nmol/L) and 55.6% achieved the sufficiency level (50 nmol/L). Only 12.1% of included women reached the optimal, 75 nmol/L level (Figure 1).

Higher maternal serum VD levels were associated with early visits to pregnancy care (*p* = 0.001), ART (*p* = 0.028) and advice from gynecologists (*p* = 0.049). No other socioeconomic, conception- or pregnancy-related parameters had a significant effect on serum VD levels. Besides the advice given by the gynecologist, no other advice source had an impact on the vitamin levels. Table 6 shows the correlations between measured serum VD levels and vitamin consumption.

Dietary VD intake was not associated with the measured maternal VD levels. However, prenatal vitamin and total VD intake showed a significant correlation with the measured VD levels (*p* < 0.001). The average measured serum VD levels of those women who reached the international 15 μg/day and the Hungarian 37.5 μg/day intake recommendations and above were 58.92 nmol/L and 65.31 nmol/L, respectively.

## 4. Discussion

The aim of this study was to evaluate the VD intake, serum VD levels and their potential influencing factors among pregnant women. Dietary VD intake was not sufficient to fulfil the intake criteria, and additional VD supplementation was required to approach the optimal level. Dietary VD intake did not correlate with the measured serum VD levels; nonetheless, the calculated total VD intake showed a strong correlation with the measured maternal VD serum levels: higher daily intake levels resulted in higher measured serum levels.

According to our results, dietary intake (2.08 μg) is insufficient to fulfil the VD intake criteria. This is in line with previous studies which found that the dietary micronutrient intake of pregnant women was far below the recommendations [32,33,34]. Scholl and Chen [35] and Bärebring et al. [28] calculated similarly low VD dietary intake values among pregnant women, 4.81 μg/day and 3.9 μg/day, respectively. Insufficient dietary intake suggests a poor health consciousness of pregnant women.

The majority of women (68.1%) took some kind of VD supplementation via pregnancy multivitamins; however, only quarter of them were aware of its VD content. VD knowledge was associated with information obtained from the internet. Together with other VD supplementation avenues (22.4%), 77.6% of included women took in VD with vitamin complexes. An Australian cohort study of low-risk pregnant women reported much higher supplementation practices: almost all of the included women (97.7%) took some form of VD-containing supplement during pregnancy, with 93.5% prenatal multivitamin usage and 62.6% taking VD together or without pregnancy multivitamins [36]. Low awareness about VD content was also found among Irish women, as 57.9% of those women who reported not taking any VD supplements had actually taken prenatal vitamins including VD [37]. Jensen et al. [38] also evaluated the VD intake sources in pregnant women. Their findings show similar results: 67.6% of included women used some kind of VD supplementation; however, only 36.9% used a product which contained the appropriate dose of the intake recommendation regarding VD. In Hungary, the dose of VD in the currently available prenatal multivitamin formulas is sufficient; it varies within the wide range of 5–25 μg per dosage. However, if the dosage is not adjusted according to personal needs and characteristics, the intake can be insufficient. It was proven by randomized controlled trials that supplementation with lower doses (10–15 μg, the international recommendations) may prevent VD deficiency but may not be enough to achieve sufficiency level [39,40,41]. Advice from a gynecologist, previous preterm birth, infertility treatment and non-spontaneous conception were associated with supplementary VD intake. On the other hand, Lee et al. [42] did not find a statistical connection between a past history of subfertility or preterm birth and VD supplementation.

According to our results, the average total VD intake was 19.27 μg/day, and only 44.5% and 12% of included women reached the international (15 μg/day) and the Hungarian (37.5 μg/day) intake recommendations, respectively; however, a higher total VD intake was observed compared to previous studies [38]. Higher total VD intake levels were observed in cases of higher educational degree, planned pregnancy and medical history of previous miscarriage. Higher educational degree was found to be associated with VD intake and supplement use in other studies, as well [43,44,45]. Other studies suggest that advanced maternal age and first pregnancy are associated with higher total VD intake levels [38,43,44,46]; however, we could not find any significant correlation regarding maternal age, first pregnancy and total VD intake. Our findings suggest that higher degree level, pregnancy planning, and previous miscarriage are associated with a higher health-consciousness of the women, which is in line with the results of previous studies [47,48].

Measured serum VD levels reflect the insufficient VD intake of included women: according to our results, 87.9% of included women did not reach the threshold of the optimal level (75 nmol/L), and 44.4% did not reach adequate (50 nmol/L) VD serum levels. Moreover, supplemental and total VD intake showed a correlation with the measured serum VD levels, which was observed in previous studies as well [49]. Despite the results highlighting the risk of low VD serum levels during the childbearing period, the condition is not uncommon among mothers. Several studies found that the maternal serum VD levels did not reach the acceptable level [40,50,51,52,53,54]. Our relatively high prevalence of VD insufficiency (44.4%) was comparable to the values found in some European countries (45% in Belgium, 35% in the UK, 44% in the Netherlands) or Australia (48%) [15].

Evaluating the relationship between the average daily VD intake (19.27 μg) and average serum VD level (52.81 nmol/L), a clear effect can be observed. Our findings are in line with the EFSA calculation: that in the case of a 15 μg per day intake, the majority of the population may achieve a 50 nmol/L serum VD concentration [8], but this intake amount is barely enough to acquire the optimal level, the lower threshold of the Hungarian normal range. This was also supported by the Endocrine Society, as a daily intake of at least 37.5–50.0 μg vitamin D intake can result in a 75 nmol/L serum 25(OH)D level [55].

Considering the overall impact of low VD status on maternal and fetal outcomes, prenatal maternal VD screening would be of high importance. In a randomized controlled trial, Rostami et al. [56] revealed that the proactive screening of VD and subsequent supplementation substantially improved birth outcomes: the incidence of preeclampsia, gestational diabetes, and preterm birth decreased by 60%, 50%, and 40%, respectively. Hungarian consensus recommendations also emphasize the pre-conceptional measurement of VD, especially in cases of infertility or recurrent miscarriages [12]. Moreover, the Australian perinatal practice guideline includes a management flowchart for clinicians to guide the management of pregnant women who are at risk of VD deficiency with screening criteria, serum sampling, supplementation, and follow-ups [57].

Our study had some limitations, as well. We analyzed cases and controls together, and instead of premature birth as a primary outcome, we used VD intake as a secondary outcome in the present analysis. The original case–control sample does not represent the general population; however, participation was offered for all eligible women, and the final decision was given by the included ones. Limitations of this study include a potentially small sample size, which may limit the generalizability of the findings; however, it is notable that every second preterm delivery at the study institution was included in the analyses. As the control group was adjusted by age and date of delivery to the case group, the women included in the term delivery group may not accurately reflect the broader population, which could influence the applicability of the recommendations. The VD dietary and supplemental intake data during pregnancy were collected after delivery, and therefore the retrospective and self-administered questionnaire-based form of the study could influence the answers; this bias can originate from the selective memory, or it is possible that respondents reported more positive behaviors during pregnancy because of social desirability. The study was run in a single institution, although this institute has a regional responsibility, which increases the generalizability of our findings. Traditional Hungarian cuisine contains little VD and is unable to provide the daily required dietary intake. Therefore, differences in dietary habits and food availability and the occurrence of enriched/fortified food products between countries can be another limitation of the study when comparing our results to those found in other countries—especially in Nordic countries. Another limitation of this study is that sun exposure was not considered in the analysis. Despite these limitations, this study provides a complex analysis about the VD intake and serum levels of pregnant women.

Our study suggests that despite the compulsory pregnancy care system in Hungary, in the health consciousness of pregnant women, VD intake levels remain below the expected level. Although the majority of included women reported the intention of more dietary-conscious behavior during pregnancy, none of the included women approached the adequate level of dietary VD intake. Poor knowledge about supplement composition also suggests low health consciousness and health literacy levels among the study population. The role of healthcare professionals is crucial in order to improve the health consciousness of pregnant women and therefore to increase their health status. As per our results, an optimal VD intake level would contribute to optimal serum levels as well, and therefore it highlights the importance of micronutrient consumption. A healthy, balanced diet rich in dietary products, fish, healthy grains, vegetables and fruits should be promoted, together with supplementary intake of the most important micronutrients, with a well-defined dose recommendation. Filling in a simple FFQ during the regular pregnancy care sessions and evaluating the dietary supplement consumption of pregnant women would provide a fair estimation of the current VD level of mothers, and therefore hypovitaminosis can be prevented.

## 5. Conclusions

During the past two decades, several studies have highlighted the importance of sufficient intake of VD and the maintenance of normal VD levels during pregnancy. Maternal dietary micronutrient intake is usually not enough to reach the optimal micronutrient intake levels. Despite the existing health-related evidence of the benefits of VD intake during pregnancy and the continuously growing dietary supplement market, the reported micronutrient intake and the measured levels are still under the recommendations. Moreover, the existing recommendations may underestimate the necessary VD intake dose to reach the normal or optimal serum level.

The main objective of this research was to assess VD consumption and blood serum levels and to identify factors that might affect these measures in pregnant women. The study found that dietary intake of VD alone was inadequate for meeting recommended intake levels, necessitating additional VD supplementation to achieve desirable concentrations. While dietary vitamin D consumption did not have a direct relationship with serum VD concentrations, the combined total of dietary and supplemental VD intake did exhibit a significant positive correlation with serum levels in pregnant women; higher cumulative intakes were associated with elevated serum concentrations.

## Figures and Tables

**Table 1:** Characteristics of pregnant women (N = 300).

Characteristics	*n*	%
Demographic characteristics		
Age group (years)		
≤24	34	11.3
25–34	158	52.7
≥35	108	36.0
Education		
primary	25	8.5
secondary	97	33.0
higher-level vocational training	50	17.0
university	122	41.5
Relationship status		
single	7	2.4
in relationship	290	97.6
Residence		
capital city, county town	134	45.0
city	89	29.9
village	75	25.1
Conception-related characteristics		
Use of contraceptives		
yes	128	43.5
no	166	56.5
Infertility treatment		
yes	35	11.8
no	261	88.2
Mode of conception		
spontaneous	287	96.3
assisted reproductive techniques	11	3.7
Pregnancy-related characteristics		
Planned pregnancy		
yes	235	80.8
no	56	19.2
First pregnancy		
yes	124	41.6
no	174	58.4
Timing of first visit at pregnancy care		
≤12 weeks	241	83.7
>12 weeks	47	16.3
Advice regarding pregnancy		
yes	242	84.0
no	46	16.0
Previous miscarriage (if not first pregnancy)		
yes	69	39.4
no	106	60.6
Previous preterm delivery (if not first child)		
yes	29	17.0
no	142	83.0

**Table 2:** Dietary vitamin D intake of women.

Food	Minimum Intake (μg/day)	Maximum Intake (μg/day)	Mean (SD) (μg/day)
Fish	0.00	4.47	0.60 (1.04)
Milk	0.00	3.00	0.80 (0.82)
Yoghurt	0.00	1.90	0.13 (0.32)
Margarine	0.00	3.20	0.55 (0.70)
Total	0.00	8.23	2.08 (1.60)

**Table 3:** Supplemental vitamin D intake with prenatal vitamins according to the characteristics of women.

Characteristics	Prenatal Vitamin Consumption (Yes)			
	*n* (%)	*p* Value *	Odds Ratio (95% CI)	*p* Value ***
Individual characteristics				
Age group (years)		0.077		
≤24	18 (54.5)		1.00	
25–34	115 (73.7)		2.34 (1.08–5.06)	0.031
≥35	72 (66.7)		1.67 (0.75–3.68)	0.207
Education		0.288		
primary	14 (58.3)		1.00	
secondary	63 (65.6)		1.36 (0.55–3.40)	0.506
higher-level vocational training	33 (66.0)		1.39 (0.51–3.77)	0.522
university	91 (74.6)		2.10 (0.85–5.20)	0.110
Relationship status		0.366 **		
single	4 (57.1)		1.00	
in relationship	200 (69.7)		1.72 (0.38–7.87)	0.482
Residence		0.771		
capital city, county town	95 (70.9)		1.24 (0.68–2.28)	0.484
city	60 (68.2)		1.09 (0.57–2.11)	0.791
village	49 (66.2)		1.00	
Conception-related characteristics				
Use of contraceptives		0.274		
yes	92 (72.4)		1.33 (0.80–2.20)	0.274
no	109 (66.5)		1.00	
Infertility treatment		0.022		
yes	30 (85.7)		3.00 (0.80–2.20)	0.028
no	172 (66.7)		1.00	
Mode of conception		0.020 **		
spontaneous	192 (67.6)		0.00 (0.00–0.85)	0.020
assisted reproductive techniques	11 (100.0)		1.00	
Pregnancy-related characteristics				
Planned pregnancy		0.219		
yes	165 (70.5)		1.48 (0.80–2.72)	0.212
no	34 (61.8)		1.00	
First pregnancy		0.112		
yes	91 (74.0)		1.51 (0.91–2.51)	0.113
no	113 (65.3)		1.00	
Timing of first visit at pregnancy care		0.020		
≤12 weeks	172 (71.7)		2.13 (1.12–4.05)	0.022
>12 weeks	25 (55.6)		1.00	
Advice regarding pregnancy		0.139		
yes	173 (71.8)		1.64 (0.85–3.15)	0.141
no	28 (60.9)		1.00	
Previous miscarriage (if not first pregnancy)		0.946		
yes	45 (65.2)		0.98 (0.52–1.85)	0.946
no	69 (65.7)		1.00	
Previous preterm delivery (if not first child)		0.376		
yes	21 (72.4)		1.49 (0.62–3.60)	0.379
no	90 (63.8)		1.00	

* Results based on Pearson’s chi-squared test, ** Results based on Fisher’s exact test. *** Results based on univariable logistic regression; 95% CI: 95% confidence interval.

**Table 4:** Overall supplemental vitamin D intake according to the characteristics of women.

Characteristics	Overall VD Supplementation (Yes)			
	*n* (%)	*p* Value *	Odds Ratio (95% CI)	*p* Value ***
Individual characteristics				
Age group (years)		0.047		
≤24	21 (63.6)		1.00	
25–34	126 (82.4)		2.67 (1.17–6.07)	0.019
≥35	81 (75.0)		1.71 (0.75–3.94)	0.204
Education		0.049		
primary	14 (60.9)		1.00	
secondary	70 (74.5)		1.88 (0.72–4.88)	0.198
higher-level vocational training	37 (74.0)		1.83 (0.64–5.22)	0.259
university	103 (84.4)		3.49 (1.32–9.19)	0.012
Relationship status		0.182 **		
single	4 (57.1)		1.00	
in relationship	223 (78.5)		2.74 (0.60–12.58)	0.194
Residence		0.098		
capital city, county town	110 (82.7)		2.06 (1.05–4.04)	0.035
city	66 (75.9)		1.36 (0.67–2.73)	0.395
village	51 (69.9)		1.00	
Conception-related characteristics				
Use of contraceptives		0.086		
yes	104 (82.5)		1.66 (0.93–2.95)	0.088
no	120 (74.1)		1.00	
Infertility treatment		0.032 **		
yes	32 (91.4)		3.50 (1.04–11.82)	0.044
no	192 (75.3)		1.00	
Mode of conception		0.076 **		
spontaneous	215 (76.5)		0.00 (0.00–1.34)	0.076
assisted reproductive techniques	11 (100.0)		1.00	
Pregnancy-related characteristics				
Planned pregnancy		0.039		
yes	186 (80.2)		1.97 (1.03–3.77)	0.041
no	37 (67.3)		1.00	
First pregnancy		0.034		
yes	102 (83.6)		1.88 (1.04–3.37)	0.035
no	125 (73.1)		1.00	
Timing of first visit at pregnancy care		0.018		
≤12 weeks	191 (79.9)		2.27 (1.14–4.54)	0.020
>12 weeks	28 (65.1)		1.00	
Advice regarding pregnancy		0.032		
yes	193 (80.4)		2.12 (1.06–4.28)	0.035
no	29 (65.9)		1.00	
Previous miscarriage (if not first pregnancy)		0.676		
yes	51 (75.0)		1.16 (0.58–2.33)	0.676
no	75 (72.1)		1.00	
Previous preterm delivery (if not first child)		0.427		
yes	23 (79.3)		1.48 (0.56–3.91)	0.429
no	101 (83.5)		1.00	

* Results based on Pearson’s chi-squared test, ** Results based on Fisher’s exact test. *** Results based on univariable logistic regression; 95% CI: 95% confidence interval.

**Table 5:** Influencing factors of total vitamin D intake (μg/day).

Characteristics	Total VD Intake (μg/day)	
	Median (IQR)	*p* Value *
Individual characteristics		
Age group (years)		0.116
≤24	7.98 (2.38–18.51)	
25–34	13.53 (5.67–24.27)	
≥35	12.69 (4.38–22.60)	
Education		0.026
primary	10.04 (3.99–17.87)	
secondary	11.73 (3.13–20.45)	
higher-level vocational training	5.74 (1.49–25.30)	
university	15.25 (7.82–24.26)	
Relationship status		0.476
single	21.12 (17.00–22.51)	
in relationship	11.85 (3.52–21.96)	
Residence		0.073
capital city, county town	13.01 (5.61–23.29)	
city	12.31 (3.74–22.21)	
village	10.89 (2.60–21.08)	
Conception-related characteristics		
Use of contraceptives		0.007
yes	12.76 (2.72–24.49)	
no	11.85 (4.72–21.08)	
Infertility treatment		0.257
yes	16.80 (9.55–22.83)	
no	11.6 (2.78–21.46)	
Mode of conception		0.034
spontaneous	11.85 (3.36–21.12)	
assisted reproductive techniques	26.43 (13.16–67.53)	
Pregnancy-related characteristics		
Planned pregnancy		0.075
yes	13.26 (3.87–22.60)	
no	9.03 (3.17–20.56)	
First pregnancy		0.124
yes	14.89 (6.08–23.84)	
no	11.68 (3.45–22.52)	
Timing of first visit at pregnancy care		0.134
≤12 weeks	12.76 (3.96–22.68)	
>12 weeks	8.23 (2.58–14.10)	
Advice regarding pregnancy		0.455
yes	11.96 (3.75–22.02)	
no	13.26 (3.34–24.62)	
Previous miscarriage (if not first pregnancy)		0.314
yes	12.09 (3.91–23.24)	
no	12.07 (3.36–21.08)	
Previous preterm delivery (if not first child)		0.607
yes	11.73 (2.60–22.6)	
no	12.76 (20.52–6.32)	

* Results based on Mann–Whitney U or Kruskal–Wallis tests.

**Table 6:** Correlations between maternal serum vitamin D levels and its sources.

Source of VD	Correlational Coefficients	
	Spearman’s r	*p* Value
Dietary VD ^1^ intake	−0.098	0.090
Prenatal VD intake	0.258	<0.001
Other VD supplement	0.154	0.008
Total VD intake	0.274	<0.001

^1^ VD: vitamin D.

## Data Availability

The dataset used and analyzed during the current study is available from the corresponding author on reasonable request due to the fact that other secondary analysis of the whole study database is still ongoing.

## Abbreviations

VD	Vitamin D
UV	Ultraviolet
ART	Assisted reproductive treatment
FFQ	Food frequency questionnaire
US	United States
SD	Standard deviation
IQR	Interquartile range
OR	Odds ratio
AOR	Adjusted odds ratio
CI	Confidence interval
